# Creating TUIs Using RFID Sensors—A Case Study Based on the Literacy Process of Children with Down Syndrome

**DOI:** 10.3390/s150714845

**Published:** 2015-06-24

**Authors:** Janio Jadán-Guerrero, Luis Guerrero, Gustavo López, Doris Cáliz, José Bravo

**Affiliations:** 1 Research Center for Communication and Information Technologies, Universidad de Costa Rica CITIC-UCR, Ciudad Universitaria Rodrigo Facio, San José 2060, Costa Rica; E-Mails: luis.guerrero@ecci.ucr.ac.cr (L.G.); gustavo.lopez_h@ucr.ac.cr (G.L.); 2 Universidad Politécnica de Madrid, Campus de Montegancedo, Madrid 28660, Spain; E-Mail: doris.caliz.ramos@alumnos.upm.es; 3 MAmI Research Lab, Universidad de Castilla La Mancha, Ciudad Real 13071, Spain; E-Mail: Jose.Bravo@uclm.es

**Keywords:** tangible user interface, RFID, literacy, Down syndrome, interaction

## Abstract

Teaching children with intellectual disabilities is a big challenge for most parents and educators. Special education teachers use learning strategies to develop and enhance motivation for complex learning tasks. Literacy acquisition is an essential and life-long skill for a child with intellectual disabilities. In this context, technology can support specific strategies that will help children learn to read. This paper introduces a Tangible User Interface (TUI) system based on Radio Frequency Identification (RFID) technology to support literacy for children with Down syndrome. Our proposed system focuses on the integration of RFID tags in 3D printed objects and low cost toys. The paper describes the experience of using some materials covering the tags and the different problems related to the material and distance of radio wave propagation. The results of a preliminary evaluation in a special education institution showed that the system helps to improve the interaction between teachers and children. The use of a TUI seems to give a physical sensory experience to develop literacy skills in children with Down syndrome.

## 1. Introduction

An appropriate education from an early stage in their life is critical to ensuring the integration of anyone into society. Literacy development is a key factor in this. Literacy starts at home and continues both in school and throughout life. In the case of children with Down syndrome, literacy development does not follow the traditional path (*i.e.*, mild or moderate Down syndrome causes difficulties in the reading and writing learning processes) [1]. The lack of reading and writing abilities may cause difficulties in their future everyday activities, such as using public transportation or buying groceries [2].

Several studies report that children with Down syndrome can develop reading and writing abilities for concrete daily activities [1,2,3,4]. Moreover, it is possible to enhance speaking, reading, listening, and writing skills with the help of specific literacy methods [4,5].

In [4] the authors proposed a literacy method designed for children with Down syndrome. The method is based on a Global Method [2], which teaches children to read and recognize words as a whole, rather than breaking the word into individual letters or groups of letters. The method incorporates words and pictograms that are repetitively shown to the children.

Regardless of their condition, children are active learners who utilize all of their senses while exploring the world around them [5]. The incorporation of technology has transformed how children interact with and learn about their environment [6]. It has also impacted the way teachers create new teaching and learning strategies using computers, tablets, and other emerging technologies [7].

Designing technological interfaces to be used by children with disabilities is not an easy task. The main difficulty is the population heterogeneity both between different disabilities and within people who have the same disability [2]. Several academic researchers have focused their efforts on the design of technology made for children with disabilities [7,8,9,10] and the use of Tangible User Interfaces (TUIs) is one of the main trends in these technological tools.

Researchers agree that TUIs to improve children’s cognitive and motor skills through computational devices. Moreover, the use of TUIs provides interaction with objects of various textures, shapes, sizes, and colors, which helps engage the child in the learning process [11,12,13].

This paper presents a TUI-based system which incorporates RFID technology. The TUIs were created using 3D printing and low cost toys. Our system is based on a literacy method for children with Down syndrome proposed by Troncoso and Del Cerro [4]. Moreover, we used a crowdsourcing approach to the creation of material and stored digital information in a Learning Objects Metadata (LOM) repository [14]. This information can be accessed by a Graphic User Interface (GUI) or printed in paperboard cards. This feature allows any teacher to both create materials and share them. The GUI and TUI prototypes were developed with the support of five special education teachers.

We performed two evaluations, testing the RFID technology with TUIs made of different materials (e.g., plastic, rubber, metal, and cardboard) in order to assess if the materials had any significant effect on RFID performance. The system was assessed in an experimental phase, conducted with five teachers and twelve children with Down syndrome.

The rest of this paper is organized as follows: Section 2 presents a background on literacy method for children with Down syndrome, TUI systems, RFID technology and related work of TUI systems for literacy. Section 3 describes the design and development strategies of our proposal. Section 4 examines the process to integrate RFID tags in objects and the evaluation with different materials. Section 5 describes the system evaluation in a real context. Section 6 provides a discussion of the results and gives some guidelines to enhance the use of our TUI system. Finally, Section 7 presents the conclusions and possible future extensions.

## 2. Background and Related Work

There are many forms of intellectual disabilities; some common examples include: Down syndrome, autism, dementia, and Traumatic Brain Injury (TBI). Clinical diagnosis may also include less severe cognitive conditions such as dyslexia (an inability to read properly), dysgraphia (a difficulty with syntax), dyscalculia (an inability in math reasoning), and Attention Deficit Disorder (ADD, a difficulty with sustaining attention).

Some of the disabilities mentioned above are characterized by significant limitations both in intellectual functioning and in adaptive behavior. Intellectual functioning involves attention problems, memory deficit, slow learning rate, language-use difficulties and lack of motivation. Adaptive behavior involves self-care, daily living skills, social development and challenging behavior [15].

Teaching reading skills to children with intellectual disabilities is a challenge for teachers because learning abilities vary greatly depending on the intellectual disability. Additionally, differences between children who have the same disability can be vast [2,8,10,16].

Previous works suggest that early reading activities encourage the progress of children with intellectual disabilities [1,3,17]. Moreover, there is a trend in the development of methods and innovative interactive technologies for literacy acquisition of children with Down syndrome [4,5,6,7]. The following subsections describe a method for teaching reading to children with Down syndrome, introduce TUIs and RFID technologies and relate these concepts.

### 2.1. Literacy Method for Children with Down Syndrome

Children with Down syndrome typically learn to read the same way as all children. The difference is that they learn at a slower rate and their instruction must match this pace. They also need to develop comprehension and retention skills [1,2,3].

Troncoso and Del Cerro proposed a literacy method designed for children with Down syndrome [4] in which children recognize the meanings of written symbols by association, the same way they do with logos or brands. This method focuses on the development of many cognitive skills, including: attention and concentration; association of words and symbols with objects; perception and discrimination; identification of similarity and difference; classification of objects to see order or relation; and developing concepts, such as space, size, and shape [2].

**Figure 1:**
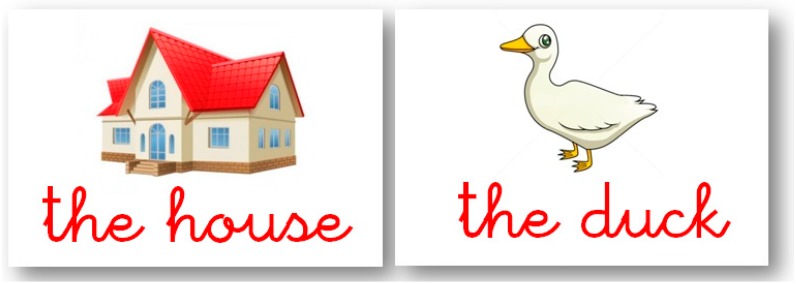
Troncoso and Del Cerro’s method cards.

The method begins with vocabulary that the children understand and are familiar with (e.g., animals, food, toys, and favorite places). Moreover, it utilizes pictograms and the written word on 15 × 10 cm cards. The font of the letters on the card is Script MS Bold, the color is red, and it is printed as large as possible. Figure 1 shows two examples of cards.

Reading activities begin by using cards in different ways, such as picture-to-picture (matching two pictures), word-to-picture (matching to make sure the child understands what he/she is reading), and word-to-word (matching the phonetics of the word). Teachers use all of these cards to develop memory, attention, association, discrimination, and denomination skills. While the method has complex resources, our research focuses only on the flashcards for reading.

### 2.2. Tangible User Interface

Ulmer and Ishii define TUIs as systems that use physical artefacts for representing and controlling digital information [11]. TUIs rely on a balance between physical and digital representations. Moreover, they help empower collaboration among children. TUIs facilitate learning and decision making through digital technology by taking advantage of our human ability to grasp and manipulate physical objects and materials [12].

TUIs take advantage of naturally acquired and developed physical object manipulation skills. Interacting with TUIs instead of traditional user interfaces also reduce the user’s cognitive effort when interacting with a system [10].

These facts led us to believe that, since children learn a variety of skills by playing with physical objects, TUIs would be a “natural” form that could be used to interact with computers. Furthermore, TUIs have great potential to be applied to education [18,19]. Our proposal uses TUIs to help children with Down syndrome develop reading abilities by encouraging them to use their sensorial and cognitive capacities.

### 2.3. Radio Frequency Identification

Radio Frequency Identification (RFID) is an automatic identification technology that transmits the identity (ID) of an item wirelessly by using radio waves, tags and readers. When a tag passes through a field covered by a reader, it transmits a unique serial number. A tag can be passive or active. Both active and passive RFIDs use radio frequency energy to communicate between a tag and a reader; however, the method of powering the tags is different. Active RFID uses an internal power source (battery) within the tag to continuously power it. Passive RFID relies on radio frequency energy transferred from the reader to the tag to power the tag. [20,21]. Our system’s implementation uses passive RFID tags.

Passive RFID tags are inexpensive (approximately $0.20 USD each) and their storage capacity is low (less than 256 bytes). The major advantage of passive RFID tags is their size, as they can be as small as 0.705 inches—approximately the size of an American dime. However, the use range of passive tags is small; depending on the tag size, it can range from a couple of inches to seven or eight inches. The main drawback of passive RFID tags is that any metal surface presents an impassable barrier [22].

The operating frequency of an RFID system directly influences its read range. Passive RFID requires stronger signals from the reader, and the signal strength returned from the tag is constrained to very low levels. RFID operation frequencies range from 125 KHz to 2.4 GHz [23]. In [24], the authors describe how interacting with different devices is beneficial to people with cognitive disabilities and present a system that use RFID technology to interact with games.

### 2.4. TUIs for Early Literacy

Several TUIs that support the educational process have been presented in literature. This section describes some of the most relevant projects.

RoyoBlocks is a system developed to allow children to build sentences. RoyoBlocks consists of 60 wooden blocks and a stuffed monkey. Each block is a word and has an embedded RFID tag. The monkey contains an Arduino powered RFID reader, which identifies the tag of each block and plays audio of the word pronunciation through a speaker. This system is offered to provide young children an opportunity to develop early literacy skills. Only preliminary testing has been performed with RoyoBlocks [9].

LIT KIT is a portable system that supports children book reading. It uses Arduino technology which communicates with Sifteo Cubes [25] to control the actions of the multi-media, architectural robot. Sifteo Cubes are blocks (based on the timeless play patterns of Legos, building blocks, and domino tiles), which communicate wirelessly and respond to each other. LIT KIT experience provides children an opportunity to interact with digital and robotic artifacts within a real, physical environment that transforms words into worlds [26].

READING GLOVE is a wearable RFID reader and a set of common objects with the tags under or inside objects. Children select an object and pick it up in order to listen the word’s pronunciation. The system engages readers in tangible and audio-based interaction [27].

TABLE TOP is a system that uses an inverted projection of a video beam. The system also uses tangible objects that are recognized by external tags and a camera. The tangible interfaces are physical objects that represent the images or words shown [7,10]. A research study describes how tabletops are an interesting approach to computer supported collaborative learning and the authors listed issues that must be addressed when using this type of interface [28].

E-DU BOX is a computer application and a tangible interface that uses a special pen-shaped mouse that vibrates according to some situations defined by the educator in the reading process. Feedback is also provided by a tangible, interactive and animated e-du agent, these agents are able to move and speak to students. All of these components use Bluetooth-based connection [29].

CO-STICAP (Stimulating Collaborative Cognitive Capabilities) is a multi-device based on distributed user interfaces and games systems. It aims to provide cognitively stimulating activities for children with Attention Deficit Hyperactivity Disorder (ADHD). It uses a laptop, a smartphone and a tangible object coded with NFC (Near-Field Communication) labels [30].

TICLE (Tangible Interface for Collaborative Learning Environments) is a platform that uses computer vision techniques to track objects and map their movements into a graphical user interface. Children interact with concrete mathematical games, such as Hanoi Tower and Tangram, while the system maps their actions to the computer screen [31].

I/O Brush is a drawing tool, in the shape of a common paintbrush, with an embedded camera and touch sensors. This device allows users to capture colors and textures of surfaces and reproduce them on the drawing canvas (consisting of a large touchscreen and a back projection screen) [32].

All the systems mentioned above offer an environment to practice the educational concepts of learning-by-doing, which can enhance the efficiency of a child’s learning process [17]. This information has allowed us to propose a system reflecting needs in our local context.

## 3. Tangible User Interface Design and Development Strategy

This section describes the design and development strategy we followed to build our proposed TUI system. The development processes was supported by five special education teachers. We used an Iterative User-Interface Design to develop prototypes and to consider some usability metrics, such as, easy to learn, efficient to use, easy to remember and pleasant to use [33]. We worked with teachers in exploring the use of the resources of the literacy method and how to adapt the technology to their needs.

The first prototype was based on the cards that the literacy method applies. We gathered the opinion of teachers and selected some concepts of study divided into four categories (fruit, animals, home and entertainment). We designed and printed a set of six cards for each category.

For the second prototype we designed a graphical user interface (GUI) running on web browser which could be accessed by computers o mobile devices. The user interface was designed to be intuitive and easy to configure for non-technical users. The information was organized in a database and used to create learning objects based on the standard IEEE Learning Objects Metadata (LOM) [14].

**Figure 2:**
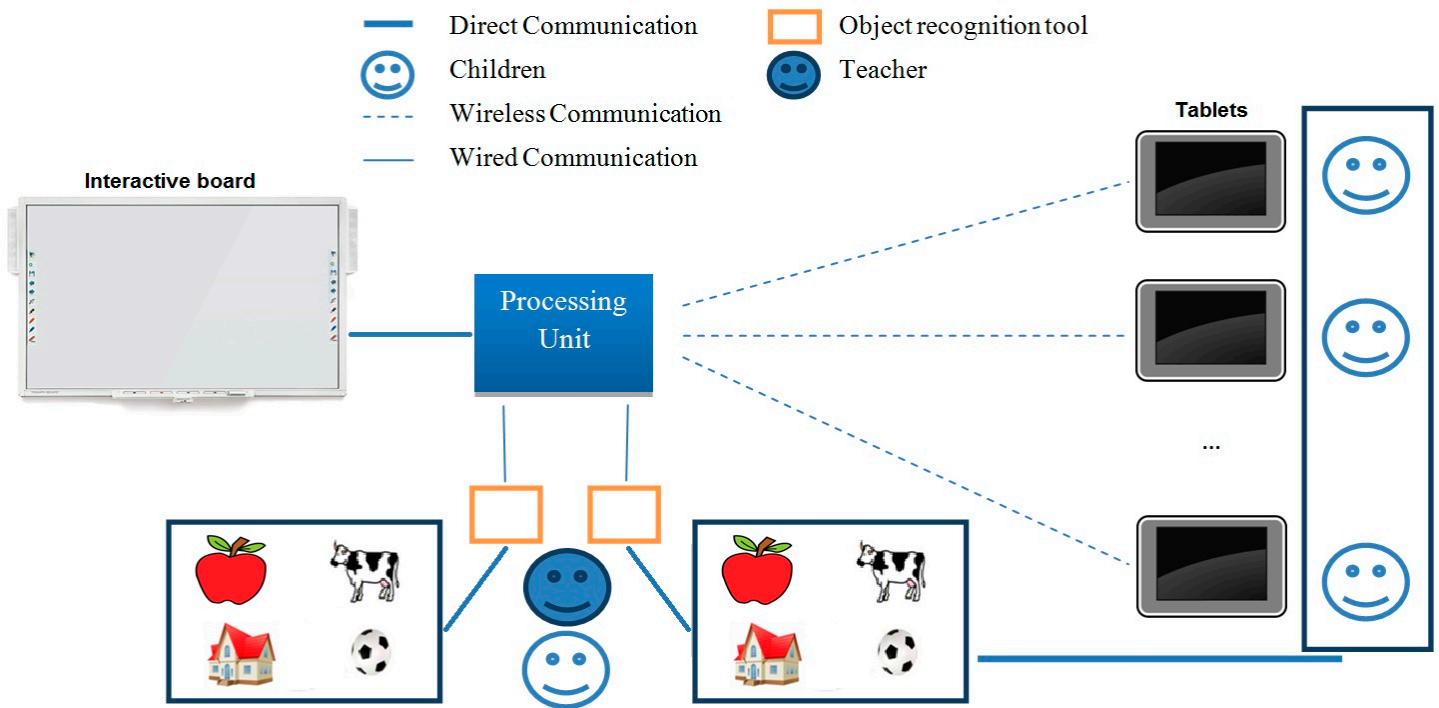
Diagram of the Tangible User Interface based on RFID sensors.

The third prototype based on TUI was the extension of the above prototypes. We analyzed the possibility to build the objects with a printer 3D and low cost toys. Next section details the process to integrate RFID technology to the objects. The backend of the system was developed using PHP and MySQL technology, and the frontend was developed using PHP, HTML5, CCS3 and JavaScript. This open-source technology allows the information to be shown in any browser of any device that is connected to the Internet. A Web service was developed to communicate with the software that identifies the tags. On the client side, the process unit was developed using Microsoft .Net technology. Figure 2 shows an architectural diagram of our system.

Three main modules compose the system:

1. The objects recognition tool. The tool has two RFID readers attached to the processing unit. It recognizes the RFID tag inside the object placed over it and sends a signal to display the associated word and pronunciation.
2. The recognizable objects set. We have several sets of 24 objects. These objects were redesigned in order to contain an RFID tag that recognizable by the system.
3. The graphic user interface. Tablets, smart phones, PC or even an interactive board with access to the Internet can be used to display the pictograms, words and pronunciations representing the physical objects.

The processing unit receives the ID for one of the RFID sensors. The software matches the received ID with the records in the repository through a Web service. The system not only displays a picture or pictogram representing the object but also presents the word and pronunciation associated with the object in each device with an open session.

The processing unit has an administration module to configure the number of sessions and the name or number that identifies the teacher and children. Tablets or mobile devices can be connected via Web through the session number. The system registers information about the activities carried out by teachers and children, such as: the start and end time of each session, the number of objects selected by the teacher and the child, and the time between each identification step.

Our design of TUIs and interaction allows pairing between either one TUI set and one GUI or, one TUI set and multiple GUIs. This allows for simultaneous interaction. Moreover, as we used Mobile-Web technology in the development process, computers, tablets or interactive boards could be used to display the digital representation of recognized objects. This last feature was designed to be used in therapy sessions (teacher-child) or in a classroom.

In order to evaluate the design of the prototypes we carried out we conducted a thinking-aloud test [34] followed by an interview about the user experience with five special education teachers. The goal was to understand their experience while teaching children with Down syndrome. This includes the method, type of educational resources and teaching strategies they used to teach children how to read. For this purpose, we explained how each prototype works and asked them to verbalize their thoughts, simulating a real scenario. The test helped us to identify some problems related to the size of pictograms, the type and color of the words, the type of voice and the time for automatic pronunciation. The feedback of the test enabled us to improve the system with the following features:

(a) The color of the physical object are the same of the pictogram in the GUI.
(b) The color of the words in cards and GUI are red.
(c) The size of the pictogram and the words are big as possible.
(d) Each word is preceded by the article.
(e) The GUI has an option to change the font, color and size of the words.
(f) The voice of the system is female.

**Figure 3:**
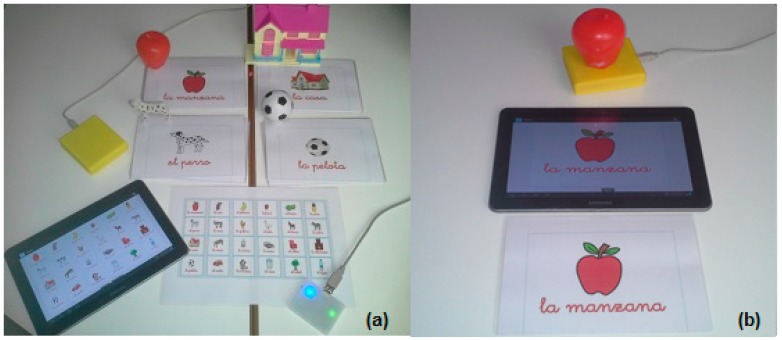
(**a**) Tangible User Interfaces for literacy; (**b**) Three interfaces (TUI, GUI and Card).

Figure 3 shows the final version of the system.

Our system differs from the solutions presented in the related work section because we used Wi-Fi technology. This feature enables long distance communications and portability. It also differs because we avoided using external tags or signs that modify the real appearance of the objects. We accomplished this by applying a digitalization and rapid manufacturing process (*i.e.*, 3D scanning and printing to redesign the objects and embed the required tags to the toys) [21].

In the interviews about user experience, all teachers agreed that the system was intuitive and easy to use. Teachers pointed out that children’s intellectual development takes place not only with the support of more capable peers or adults through social interaction, but also with the evolvement of social and cultural artifacts. This assertion is based on pedagogical theories studied by Vygotsky [35]. In this sense the interaction with technology could help to develop strategies for connecting abstract and concrete representations of concepts.

## 4. Integrating RFID Tags into Objects

**Figure 4:**
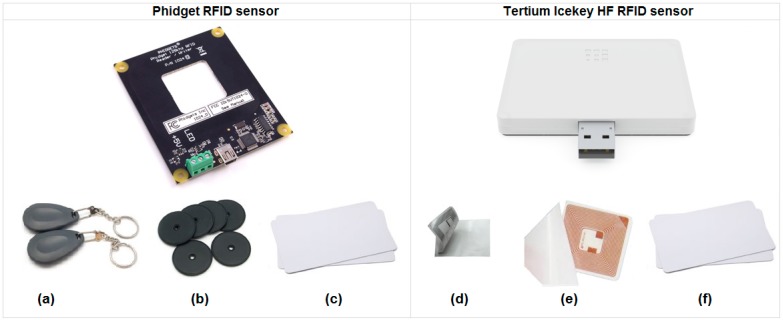
Passive RFID tags of Phidget RFID and Tertium icekey Hf readers. (**a**) ABS Key Fob Blue; (**b**) Clothing Button: 2.5 cm diameter; (**c**) Credit Card: 4 × 3 cm; (**d**) HF tag Sticker: 1.8 × 3.6 cm; (**e**) HF tag Sticker: 4.3 × 4.3 cm; (**f**) Credit card: 4 × 3 cm.

This section describes the process to integrate RFID tags into the tangible objects. We selected two RFID readers. The first was Phidget RFID reader [36] which has a set of three tags: (a) ABS Key Fob Blue; (b) Clothing Button-2.5 cm diameter; (c) Credit Card 4 × 3 cm. The second was Tertium icekey Hf reader [37] with a set of three tags: (d) HF tag Sticker 1.8 × 3.6 cm; (e) HF tag Sticker 4.3 × 4.3 cm; (f) Credit card 4 × 3 cm (see Figure 4). Each tag relies on a small chip, which is implanted in it and has a unique code of identification (ID). The tag can be attached or embedded to any kind of things. Then, an electronic scanner (RFID reader) uses radio signals to read or track the ID tag [20].

According to the manufacturers, passive tags require a strong RF field to operate and their effective range is limited to an area in close proximity to the RFID reader. The distance over which the RFID tag is usable is affected by things, such as: the tag shape and size, the materials being used in the area near the reader, and the orientation of the reader and tag with respect to each other. The smaller a tag is, the closer it must be to the reader to be detected [38]. As a result of declaring that the recognizable object could not have external tags as a key characteristic of our system, we considered two main aspects: the material of the object and the orientation of the tags.

**Figure 5:**
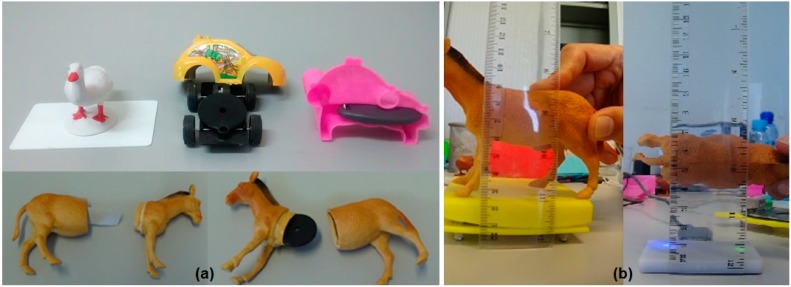
(**a**) Tags in toys and 3D printed objects; (**b**) Measure process with Tertium, icekey, Hf and Phidget RFID readers.

We created 3D printed objects with internal space for tags, and used low cost toys that could be disassembled. Figure 5a shows some toys with the embedded tags. Figure 5b shows the measuring process with the objects.

**Figure 6:**
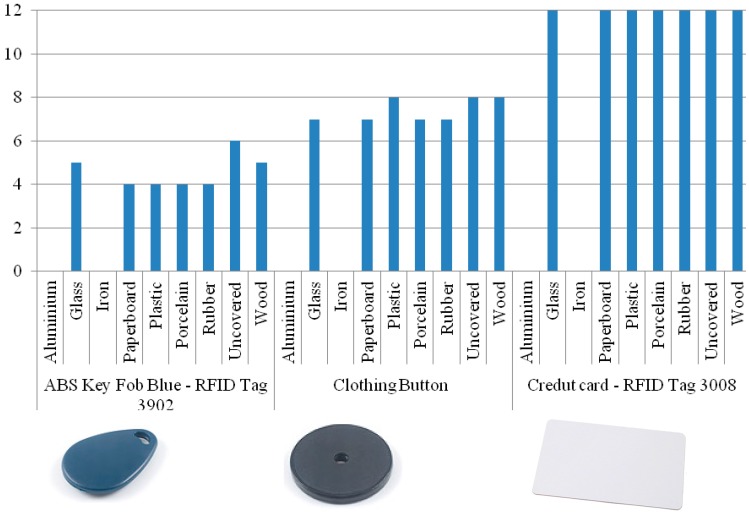
Distances registered using Phidget RFID sensor.

**Figure 7:**
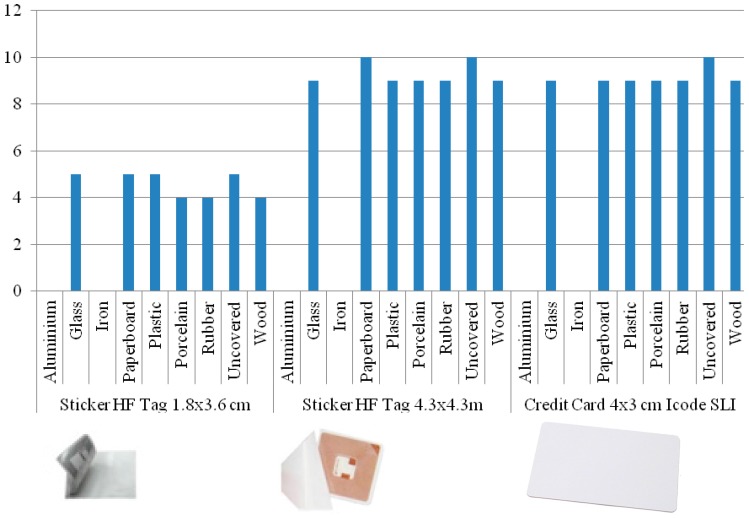
Distances registered using Tertium Icekey HF RFID sensor.

In order to measure how the effectiveness of the readers was affected by materials, we carried out a test with the following materials: aluminum, glass, iron, paperboard, plastic, porcelain, rubber and wood. The results of the measurements are shown in Figure 6 and Figure 7.

Based on the functionality of passive RFID tags [39,40] the measures were performed in such a way that the reader and the tag were parallel to each other. The three tags of each reader were placed inside or over each material and we performed two measurements for each one

One revised study mentioned that RFID transmits signals through electromagnetic waves; therefore, it is extremely sensitive to metals, liquids or shiny covers [41]. Our results confirmed this fact—Figure 6 and Figure 7 show that metallic surfaces were not recognized by any of our readers. We also confirmed that the distance of recognition is directly proportional of the size of the tags [42]. The base line was established testing uncovered tags, and they registered the maximum distance of 12 cm for Phidget and 10 cm for Tertium.

The results were similar for both readers. We noted that the presence of some materials, for example rubber, near tags can degrade the performance of the RFID, preventing them from being read. However, with glass, paperboard, plastic, porcelain and wood the measurements were constant. We observed that the position also influences in the recognition. The radio wave sensor works well when the tags are placed parallel, but the identification decreases when the tag is in a vertical position.

## 5. System Evaluation

Structured experiments were performed to assess the perceived usefulness and usability of our system in real scenarios. We carried out the evaluation in a special education institution with five teachers and twelve children with Down syndrome. To perform the evaluation, teachers were trained on how to use our system and they were who incorporated it into the learning process that they normally follow. Each child participated in two processes: (1) teacher-child, one teacher interacts with one child; (2) Self-learning, one child interacts individually with the system.

In the first scenario, the system was set up with the following components: one Laptop (Processing unit), two RFID readers, a set of 24 recognizable objects, and a tablet or interactive board. Each teacher carried out reading activities related to the method with each assigned child. The procedure was performed with three versions of prototypes: Card (paperboard cards), GUI (digital cards displayed on a tablet or interactive board) and TUI (Tangible RFID objects). The teacher selects one card and shows it to the child. The teacher read the associated word of the pictogram. The child repeats the pronunciation and identify the concept. In the GUI version the teachers works with a tablet or interactive board. The system shows a menu with the set of 24 pictograms. The teacher selects randomly one pictogram and the system reads the word. Next is the turn of the child. In the TUI version the teacher and the child interact with the set of 24 physical objects. The teacher selects randomly one tangible object and the system pronounces the name of the object when it is close of the sensor. The child repeats the task performed by the teacher.

**Figure 8:**
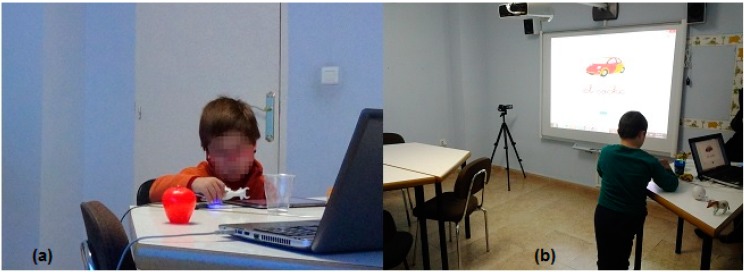
(**a**) A child interacts with the tangible objects and a tablet; (**b**) A child interacts with the tangible objects and the interactive board.

In the second scenario, each child interacts with the three prototypes by himself. The prototype is selected randomly and each child is free to use the cards, the tablet and the tangible objects during 5 min each one. This activity was designed to observe a self-learning behavior. Figure 8 shows two examples of sessions: in Figure 8a, a child interacts with the tangible objects and a tablet; and in Figure 8b, another child interacts with the tangible objects and the interactive board.

Based on Hawthorne studies we made a shift from controlled experiments toward a complex social situation [43], *i.e.*, we observed the behavior of both teachers and children with the system. The interaction with tangible objects was very natural for children. They experienced positive emotions and spent an enjoyable time with GUI and TUI.

Since the evaluation was not designed to obstruct the traditional learning process, we did not assess the children appreciation of the tool during the evaluation; however, their productivity increased, as a sign of motivation. Moreover, according to the teachers, they provided more and better feedback when using our system than in traditional lessons.

**Figure 9:**
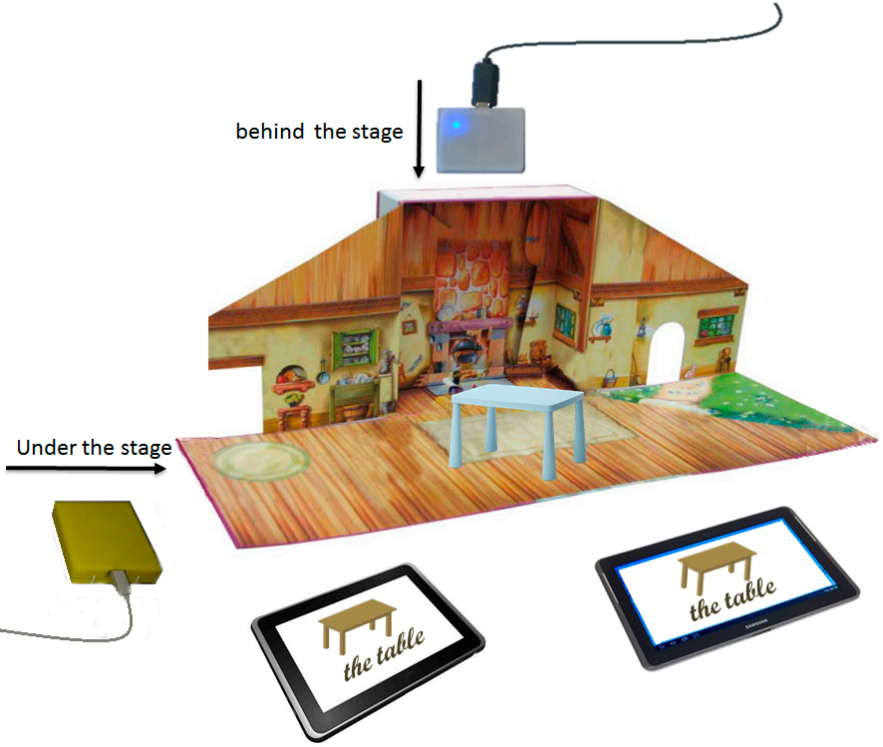
Using two sensors to facilitate object recognition.

During the evaluation, two researchers observed the progress and noticed that:

1. Children required visual or auditory confirmation of the system when an object was recognized. We implemented a beep to confirm recognition.
2. Children had difficulties with some objects, specifically 4-legged animals (e.g., cow, dog, and donkey). When the children placed them in a vertical position, the RFID tag was not close enough to be recognized. We used two readers to avoid this problem. Figure 9 shows the configuration of two RFID readers.

We observed the engagement and interaction of children with the tangible objects. This engagement may result from motivation of toys or objects which are familiar for children with Down syndrome [13]. The teachers also expressed motivation when using the system and its enhanced capabilities to create new learning and reading materials. The toys and other objects were attractive to create stories and strengthen language skills.

## 6. Discussion

After the validation process, all of the teachers agreed that the system is useful in enhancing the first stages of the literacy process. One of the teachers mentioned that the system could not only help children with intellectual disabilities, but also children with visual disabilities when touching and recognizing the objects. The teacher believed that the adaptation of novel technology is necessary for the integration of children with disabilities in the classroom.

Teachers also thought that tangible interfaces emphasize the connection between the body and cognition, facilitating thinking through physical actions. Tangible media is a complement to literacy method because it gives digital information and physical shape to the studied words.

It is important to mention that most of the participants in the evaluation process pointed the fact that the system could be used to help the literacy process for any child, including children without disabilities.

A key component of learning strategies to develop reading skills is interaction [13,16]. In this sense, we analyzed two learning strategies: teacher-child and self-learning. In each strategy we carried out a random tests during each session. Table 1 shows the results of the level of interaction with the system between teachers and children.

**Table 1:** Results of the level of interaction

Learning Strategy	Interface	Children	Teachers
Teacher-child	Card	low	high
	GUI	medium	medium
	TUI	high	Low
Self-learning	Card	low	NA
	GUI	medium	NA
	TUI	high	NA

The level of interaction children had with TUI was high in relation to that of GUI and Card. It was evident that children used not only verbal language but also the sensorial-perceptive stimulation. In the GUI, the interaction was shared between the child and the teacher. However the interaction with the traditional interface (Cards) was low by children.

In the evaluation context we observed some problems with the identification process. Some tangible objects were not easily identified because of the position of the tag. However, children learned to find the best position by moving the object until one of the readers beeped. We also observed that teachers who participated in the study had difficulties in positioning some objects. This led us to think that we could improve the identification of the objects. Based on previous works on using RFID technology in the classroom [44,45,46], we propose a stage with the two readers placed in horizontal and vertical positions, as depicted in Figure 9.

The simulation of a real stage could help the children identify the tangible objects easier. Teachers could create learning strategies to develop skills, such as: attention and concentration; associating words and symbols with objects; sight-word recognition; and phonics. Teachers could also help introduce and develop new vocabulary; perception and discrimination; identifying similarity and differences; classifying objects; seeing order or relationships; developing concepts like space, size, and shape; and exploring, manipulating, and using creative imagination [34,47].

Children with Down syndrome will usually be delayed in their language comprehension compared to other children of the same age. It is important for them to start with vocabulary they can understand and that words or objects be familiar to them. Both teachers and parents need to spend time with the children to enhance their reading skills and the system could contribute to that. Reading practice will also help develop working memory skills. The ability to read and write facilitates understanding of general knowledge from the school curriculum and supports the necessary skills for problem solving in real life.

## 7. Conclusions

In this paper, the use of RFID technology was analyzed when implementing a set of tangible objects to support literacy. Different problems related to the material and distance of radio wave propagation were evaluated.

We have shown that Tangible User Interfaces can be created from common objects in our daily environment and that this integration can be important to design new educational technologies that support literacy.

The results of the prototype evaluation showed that the system, not only assists the teacher, but also helps improve the interaction between children and non-traditional user interfaces. It is important to mention that the educational inclusion of children with intellectual disabilities works better when all of their classmates are involved in same activities. A technological system for supporting this process can be used but can never replace the teacher guide.

We found that a system with a Tangible User Interface, like the one we developed, can support teachers or parents working with children with intellectual disabilities, especially if the system supports a well-known method, like the Troncoso and Del Cerro literacy method. We want to highlight that the final users of our system are the teachers; our system supports those who are experts in teaching literacy to children with learning disabilities.

Depending on the cognitive deficit of the student, more features can be easily implemented and incorporated into the system. For instance, the card on the tablet could include a sound or video. In the same way, using their finger, children can paint over or follow the written word on the tablet to introduce writing. These new features are scheduled for a future version of the prototype and more evaluations and experiments will be conducted.

In educative systems the focus is not the technology, but the interaction it facilitates and its ultimate learning effects. This interaction can also play an important role in the learning of new skills because children learn by experiencing the environment through their senses. The use of multisensory strategies can help to scaffold literacy learning [16].

Kubicki, Lepreux and Kolski [48,49], presented TangiSense, a table that allows interactions with tangible objects. We consider that by incorporating TangiSense capabilities and our system, teacher could engage children in more complex and complete activities, and allow better monitoring of the situation at all times.
